# The effects of pre-pregnancy body mass index and gestational weight gain on perinatal outcomes in Korean women: a retrospective cohort study

**DOI:** 10.1186/1477-7827-9-6

**Published:** 2011-01-18

**Authors:** Sae-Kyung Choi, In-Yang Park, Jong-chul Shin

**Affiliations:** 1 Department of Obstetrics and Gynecology, College of Medicine, The Catholic University of Korea, 505 Banpo-dong, Seocho-gu, Seoul, Korea

## Abstract

**Background:**

The purpose of the study was to evaluate the effects of maternal pre-pregnancy body mass index (BMI) and gestational weight gain on perinatal outcomes in a population of Korean women.

**Methods:**

We retrospectively reviewed the medical records of 2,454 women who had received antenatal care at Seoul St. Mary's Hospital from January 2007 to December 2009. We used World Health Organization definitions for Asian populations of underweight (BMI < 18.5), normal (BMI equal or higher 18.5 and < 23), overweight (BMI equal or higher 23 and < 25), and obese (BMI equal or higher 25). We analyzed perinatal outcomes according to the pre-pregnancy BMI and weight gain during pregnancy, and calculated the adjusted odds ratios (ORs) and 95% confidence intervals (CIs) from multiple logistic regression models by considering maternal age, parity, number of fetuses, length of gestation, and medical history.

**Results:**

Among obese women, the adjusted ORs for gestational diabetes, hypertensive disorder, and incompetent internal os of cervix were 4.46, 2.53, and 3.70 (95% CI = 2.63-7.59, 1.26-5.07, and 1.50-9.12), respectively, and the adjusted ORs for neonatal complications such as macrosomia and low Apgar score were 2.08 and 1.98 (95% CI = 1.34-3.22 and 1.19-3.29), respectively, compared with normal weight women. However, there was no positive linear association between gestational weight gain and obstetric outcomes. In normal weight women, maternal and neonatal complications were significantly increased with inadequate weight gain during pregnancy (p < 0.0001 and = 0.0180, respectively), and we observed similar results in underweight women (p = 0.0136 and 0.0004, respectively).

**Conclusions:**

This study shows that pre-pregnancy overweight and obesity are more closely related to the adverse obstetric outcomes than excess weight gain during pregnancy. In addition, inadequate weight gain during pregnancy can result in significant complications.

## Background

Obesity has been frequently cited as a health problem in women of childbearing age. A recent report found that 25% of the adult population was obese. The obesity rate has rapidly increased in the general population and in women of childbearing age [1,2]. According to a study conducted from 2002 to 2004 in Scotland, 20% of women who received antenatal care were obese, representing a twofold increase over the past 10 years [3]. A similar study in the United States reported that the percentage of obese women who received antenatal care increased from 16% in 1980 to 36% in 1999 [4]. Gestational weight gain is also higher than ever before, with approximately 40% of pregnant women gaining more weight than is recommended [5].

Obesity during pregnancy may cause adverse outcomes, not only in the mother but also in the child. Many studies have found that gestational diabetes, preeclampsia, emergency caesarean section, postpartum hemorrhage, wound infections, preterm delivery, large for gestational age (LGA), and fetal death in utero (FDIU) were more common in obese mothers, implying that obesity during pregnancy is a major challenge for healthcare providers [6-8]. Maternal obesity may cause adverse outcomes in offspring in addition to neonatal complications. Recent studies have reported the interrelation between the pre-pregnancy weight of mothers and children's obesity that occurred before the age of 9 years [9].

Previous studies, however, mostly focused on the influence of pre-pregnancy overweight or obesity on pregnancy outcomes. Moreover, in relation to Asian women, only a few studies have evaluated the influence of pre-pregnancy BMI and gestational weight gain on perinatal outcomes. In particular, there is a paucity of studies conducted on Korean women. The expert group of the World Health Organization (WHO) suggested the use of the modified body mass index (BMI) classification in view of the fact that the prevalence of obesity was lower in Asian countries than in Western countries, but the rate of obesity-related diseases was the other way round [10], demonstrating that studies on obesity in Korean women may be helpful to evaluate the traits of pregnant Asian women. Especially, such studies would be worthy in the sense that perinatal outcomes are judged on the basis of pre-pregnancy BMI and gestational weight gain.

The objective of this study was to evaluate the effects of maternal pre-pregnancy BMI and gestational weight gain on perinatal outcomes in a population of Korea women.

## Methods

We retrospectively reviewed the medical records of women who received antenatal care at Seoul St. Mary's Hospital from January 2007 to December 2009. A total of 2,454 pregnant women were initially enrolled in this study. However, only those who had successful deliveries in our hospital were considered for this study. Women with missing information for the independent variables, BMI before pregnancy and at the time of delivery, were excluded from the analysis. The final sample consisted of 2,413 women.

Weight and height were measured directly at admission for delivery and were used to calculate gestational weight gain and pre-pregnancy BMI (maternal weight in kg/height in m^2^), while pre-pregnancy weight reported by each subject was used to calculate pre-pregnancy BMI. We used WHO definitions for Asian populations of underweight (BMI < 18.5), normal (18.5 ≤ BMI < 23), overweight (23 ≤ BMI < 25), and obese (25 ≤ BMI). Gestational age was estimated from the last menstrual period, and this was confirmed or corrected by ultrasonography.

We analyzed the influences of pre-pregnancy BMI and gestational weight gain on perinatal outcomes, and these were analyzed on the basis of maternal and neonatal complications, respectively. The following maternal complications were included: gestational diabetes, hypertensive disorder, placenta abruption, incompetent internal os of cervix (IIOC), preterm delivery, preterm premature rupture of membrane (PPROM), and other hematologic disorders. The following neonatal complications were included: FDIU, fetal anomaly, intrauterine growth restriction (IUGR), small for gestational age (SGA), LGA, meconium-stained amniotic fluid, low Apgar score, and neonatal intensive care unit (NICU) admission. The criterion for birth weight was based on the gestational age at delivery, where cases that were less than the 10^th ^percentile or more than the 90^th ^percentile were classified as SGA or LGA; this criterion was derived from a worldwide study conducted by Alexander GR et al [11]. Meconium-stained amniotic fluid was limited to third-degree meconium-stained amniotic fluid of the clinically defined thick meconium, and a low Apgar score was defined as when the 1 or 5-minute Apgar score was less than 7.

All statistical analyses were performed using SAS software (version 9.1). The continuous variables were expressed as the means ± standard deviation by analysis of the variance (ANOVA). The clinical characteristics and obstetric outcomes of the categorical data or frequency of an event were analyzed using Fisher's exact test and the chi-square test with Yates' correction, respectively. The significance level was limited to p-values less than 0.05.

To evaluate the interrelation between pre-pregnancy BMI and pregnancy risk, multiple logistic regression models were used. Maternal age, parity, number of fetuses, length of gestation, and the history of medical illness were considered as confounding factors. The models were adjusted for these factors in order to analyze the outcomes. Adjusted odds ratios (ORs) and 95% confidence intervals (CIs) were calculated.

This study was approved by an ethics committee of the Clinical Research Coordinating Center of the Catholic Medical Center.

## Results

The mean pre-pregnancy BMI was 20.9 ± 2.8 kg/m2; 417 subjects (17.3%) were underweight (BMI < 18.5), 1,556 (64.5%) were normal (18.5 ≦ BMI < 23), 236 (9.8%) were overweight (23 ≦ BMI < 25), and 204 (8.5%) were obese (25 ≦ BMI).

The clinical characteristics and outcomes of the subjects are summarized in Table 1. Overweight and obese women were significantly older than those in the normal weight group (31.3 ± 3.6, 32.3 ± 3.9, 33.4 ± 4.1, 33.3 ± 3.9 years, respectively, p < 0.0001). In the overweight and obese groups, the percentage of highly educated women was lower than that in the other two groups (89.5% vs. 89.3% vs. 82.6% vs. 80.4%, respectively, p = 0.0014), and the percentage of working women was lower (45.8% vs. 50.1% vs. 44.9% vs. 34.3%, respectively, p = 0.1112) than that in the normal group. The percentage of multigravida in overweight and obese women was higher than that in the normal group (30.9% vs. 40.0% vs. 57.2% vs. 53.4%, respectively, p < 0.0001). The rate of cesarean delivery for overweight and obese women was higher than that for the normal weight women (30.2% vs. 34.2% vs. 45.8% vs. 56.4%, respectively, p < 0.0001) (Table 1). However, pre-pregnancy BMI values did not influence a significant increase in the incidence of emergency cesarean section.

**Table 1:** Characteristics of the study population

	Underweight (BMI < 18.5)	Normal (18.5 ≤ BMI < 23)	Overweight (23 ≤ BMI < 25)	Obese (25 ≤ BMI)	p-value
N	417 (17.3)	1556 (64.5)	236 (9.8)	204 (8.5)	
Age^a^	31.3 ± 3.6	32.3 ± 3.9	33.4 ± 4.1	33.3 ± 3.9	< 0.0001
Educational status^b^ Under middle school High school College or higher	1 (0.2) 43 (10.3) 373 (89.5)	7 (0.5) 160 (10.3) 1389 (89.3)	1 (0.4) 40 (17.0) 195 (82.6)	1 (0.5) 39 (19.1) 164 (80.4)	0.0014
Occupation^b^	191 (45.8)	779 (50.1)	106 (44.9)	70 (34.3)	0.0002
Medical history^b^	43 (10.3)	153 (9.8)	25 (10.6)	32 (15.7)	0.0857
Parity^b ^(multigravida)	129 (30.9)	623 (40.0)	135 (57.2)	109 (53.4)	< 0.0001
Length of gestation^a ^(weeks)	38.7 ± 2.7	38.7 ± 2.7	38.2 ± 3.3	37.7 ± 3.8	< 0.0001
Mode of delivery^b^ Vaginal delivery Cesarean section	291 (69.8) 126 (30.2)	1024 (65.8) 532 (34.2)	128 (54.2) 108 (45.8)	89 (45.6) 115 (56.4)	< 0.0001
Weight gain^a ^(kg)	13.4 ± 4.2	13.4 ± 4.2	12.31 ± 4.91	11.0 ± 5.2	< 0.0001
Weight of fetus^a ^(g)	3033.5 ± 584.0	3122.7 ± 603.0	3109.4 ± 740.2	3069.3 ± 856.1	0.0728
Maternal complications^b^	50(12.0)	226(14.5)	52(22.0)	68(33.3)	< 0.0001
Neonatal complications^b^	137(32.9)	444(28.5)	81(34.3)	74(36.3)	< 0.0001

^a ^Based on ANOVA (mean ± SD)

^b ^Based on chi-square or Fisher's exact test (count, %)

BMI, body mass index; SD, standard deviation

### Pre-pregnancy BMI

According to the perinatal outcomes, the incidences of maternal complications were 12.0%, 14.5%, 22.0%, and 33.3%by BMI, respectively, and the incidences of neonatal complications were 32.9%, 28.5%, 34.3%, and 36.3% by BMI, respectively, all of which were significantly different (p < 0.0001).

For the maternal complications, gestational diabetes was observed in 0.2% of the underweight group, but reached 12.3% in the obesity group (p < 0.0001). Furthermore, the incidence of hypertensive disorders increased from 2.4% in the underweight group to 6.9% in the obesity group (p < 0.0005). In addition, intergroup differences were statistically significant for the incidences of preterm delivery, PPROM, and IIOC (p = 0.0463, 0.0008, and 0.0474, respectively). The risk ratio for each complication was calculated on the basis of maternal age, parity, number of fetuses, length of gestation, and medical history relative to the normal weight group. In the case of gestational diabetes, the obesity group showed a statistically significant increased risk with an OR of 4.46 (95% CI = 2.63-7.59). For hypertensive disorder, the overweight (OR 2.2; 95% CI = 1.11-4.58) and obesity groups (OR 2.53; 95% CI = 1.26-5.07) showed statistically significant increased risks. Furthermore, in relation to IIOC, the obesity group showed a statistically significant increased risk with an OR of 3.70 (95% CI = 1.50-9.12) (Table 2).

For the neonatal complications, LGA reached 4.1%, 7.7%, 14.0%, and 14.7% in the respective groups, and this was a representative item that showed significant differences according to the BMIs (p < 0.0001). Intergroup differences were observed in the indices that indirectly demonstrate fetal distress. In these cases, the incidence rates were higher not only in the overweight and obese groups but also in the underweight group than in the normal weight group. Among the indices that measure neonatal outcome, the ORs for a low Apgar score were high in the overweight group (OR 2.12; 95% CI = 1.20-3.18) and in the obesity group (OR 1.98; 95% CI= 1.19-3.29) (Table 2).

**Table 2:** Adjusted odds ratios for pregnancy risks

	Underweight (BMI < 18.5)	Normal (18.5 ≤ BMI < 23)	Overweight (23 ≤ BMI < 25)	Obese (25 ≤ BMI)
Maternal complications				
Gestational diabetes	0.09 (0.01-0.66)	1.00	1.58 (0.80-3.15)	4.46 (2.63-7.59)
Hypertensive disorder	1.08 (0.52-2.23)	1.00	2.26 (1.11-4.58)	2.53 (1.26-5.07)
Placenta abruption	0.74 (0.20-2.73)	1.00	0.29 (0.04-2.38)	0.18 (0.02-1.66)
IIOC	1.32 (0.44-3.93)	1.00	1.34 (0.41-4.38)	3.70 (1.50-9.12)
Preterm delivery	0.40 (0.19-0.86)	1.00	1.10 (0.57-2.10)	0.58 (0.26-1.28)
PPROM	1.15 (0.57-2.32)	1.00	0.95 (0.47-1.94)	1.15 (0.57-2.32)
Hematologic disorder	2.60 (0.72-9.40)	1.00	2.30 (0.46-1.65)	-
Neonatal complications				
FDIU	3.94 (0.86-18.02)	1.00	2.86 (0.46-17.62)	0.66 (0.06-7.28)
Anomaly	1.30 (0.76-2.22)	1.00	1.14 (0.57-2.30)	0.73 (0.30-1.74)
IUGR	1.36 (0.64-2.88)	1.00	0.57 (0.13-2.47)	1.83 (0.72-4.67)
SGA	1.38 (1.02-1.88)	1.00	0.69 (0.42-1.13)	1.08 (0.69-1.69)
LGA	0.46 (0.18-1.19)	1.00	1.79 (0.92-3.48)	2.77 (1.49-5.16)
Thick meconium-stained amniotic fluid	1.03 (0.61-1.72)	1.00	2.12 (1.18-3.79)	0.86 (0.36-2.03)
Low Apgar score	1.39 (0.91-2.12)	1.00	1.96 (1.20-3.18)	1.98 (1.19-3.29)
NICU admission	1.32 (0.79-2.20)	1.00	1.42 (0.78-2.61)	1.11 (0.57-2.16)

Based on multiple logistic regression models adjusted for age, parity, numbers of fetuses, pregnancy (day), and medical history

BMI, body mass index; IIOC, incompetence of internal os of cervix; PPROM, preterm premature rupture of membrane; FDIU, fetal death in uterus; IUGR, intrauterine growth restriction; SGA, small for gestational age; LGA, large for gestational age; NICU, neonatal intensive care unit

### Gestational weight gain

To ascertain the incidence of complications according to gestational weight gain, we classified gestational weight gain in subjects into the following 3 groups; (1) less-than-recommended weight gain, (2) recommended weight gain, and (3) more-than-recommended weight gain according to Institute of Medicine (IOM) guidelines. In the underweight group, the incidence of maternal and neonatal complications increased significantly higher with less-than-recommended gestational weight gain (p = 0.0136 and 0.0004, respectively). We observed similar results in the normal weight group (p < 0.0001 and p = 0.0180, respectively). In the overweight and obese groups, on the other hand, there were no significant differences between weight gain and the incidence of complications (Table 3).

**Table 3:** Obstetric outcomes according to weight gain

Weight gain during pregnancy (kg)		Under recommendation*	Recommendation	Over recommendation	p-value
Underweight (BMI < 18.5)	Maternal complications	30 (17.3)	18 (8.9)	2 (4.8)	0.0136
	Neonatal complications	75 (43.4)	54 (26.7)	8 (19.1)	0.0004
	Cesarean section	48 (27.8)	62 (30.7)	16 (38.1)	0.4150
Normal (18.5 ≤ BMI < 23)	Maternal complications	111 (21.5)	82 (11.6)	33 (9.9)	< 0.0001
	Neonatal complications	171 (33.1)	186 (26.4)	87 (26.1)	0.0180
	Cesarean section	171 (33.1)	224 (31.7)	137 (41.0)	0.0107
Overweight (23 ≤ BMI < 25)	Maternal complications	9 (31.0)	15 (19.2)	28 (21.7)	0.4006
	Neonatal complications	13 (44.8)	27 (34.6)	41 (31.8)	0.3927
	Cesarean section	12 (41.4)	35 (44.9)	61 (47.3)	0.8310
Obese (25 ≤ BMI)	Maternal complications	10 (38.5)	19 (38.0)	39 (30.5)	0.5298
	Neonatal complications	11 (42.3)	23 (46.0)	42 (32.8)	0.2231
	Cesarean section	15 (57.7)	25 (50.0)	75 (58.6)	0.5767

Based on chi-square or Fisher's exact test (count, %)

BMI, body mass index; C/S, cesarean section

*Recommended gestational weight gain; underweight: 12.5-18 kg, normal: 11.5-16 kg, overweight: 7-11.5 kg, obese: 5-9.1 kg

In the underweight group, the incidence of PPROM were maternal complications that demonstrated an inverse relationship to weight gain (p = 0.0074). Among neonatal complications, the rates of SGA, low Apgar score, and NICU admission were inversely proportional to gestational weight gain (p = 0.0016, p < 0.0001, p = 0.0014, respectively). The normal weight group showed an increased incidence of gestational diabetes, placenta abruption, preterm delivery and PPROM with less weight gain (p = 0.0392, 0.0002, 0.0225, and p < 0.0001, respectively). Furthermore, as observed in the underweight group, the incidence of neonatal complications such as SGA, low Apgar score and NICU admission was increased with less weight gain during pregnancy (p = 0.1127, and p < 0.0001, respectively). The increased incidence was observed for LGA with more weight gain in the normal weight group (p < 0.0001).

The rate of cesarean section was lower in subjects with recommended gestational weight gain in the normal weight group (p = 0.0107). Although the increased in the rate of cesarean section for arrest disorders was directly proportionate to weight gain, the rate of cesarean section for fetal distress was inversely proportionate to it.

## Discussion

Our study found that pre-pregnancy BMI was more closely related to perinatal outcomes than gestational weight gain in Korean women.

In women who were overweight or obese before pregnancy, we observed a higher frequency of gestational diabetes, hypertensive disorder, IIOC, LGA, thick meconium-stained amniotic fluid, and low Apgar score, supporting the results of previous studies. Voigt *et al*. analyzed German perinatal statistics and demonstrated higher rates of hypertension, preeclampsia, gestational diabetes, fetal macrosomia, fetal structural anomalies, and low neonatal Apgar score in obese than in normal weight women[12]. Bhattacharya *et al*., who compared 1,857 obese pregnant women with 14,076 normal pregnant women, reported that obese pregnant women had significantly higher frequencies of preeclampsia, gestational hypertension, emergency caesarean section, preterm delivery at less than 33 weeks of gestation, and birth weight over 4,000 g [13].

Adverse perinatal outcomes may occur due to not only excessively high pre-pregnancy BMI but also excessively low BMI. Murakami *et al*. concluded that pre-pregnancy BMI and perinatal outcomes showed a U-shaped interrelation. They observed that overweight and obese women were at a higher risk of cesarean section, preeclampsia, and gestational diabetes than normal weight women, but underweight women showed a higher risk of low birth weight infants, thereby elevating the rate of infant hospitalization [14]. In our study, the incidence rate also showed a U-shaped curve for the majority of neonatal outcome-related indices, except for LGA, confirming that pre-pregnancy underweight was a risk factor for neonatal complications.

Many previous studies have reported that in addition to maternal and neonatal complications, the rate of cesarean section increases in obese pregnant women [15-18]. Poobalan *et al*. conducted a meta-analysis on a cohort study performed from 1996 to 2007 and found that the risk of cesarean section was higher in overweight or obese women than in women with normal BMI. In addition, they mentioned that the risk of emergency cesarean delivery was more than elective, although both were higher with increasing BMI [18]. Many reports have indicated that the higher rate of cesarean section in obese pregnant women is due to neonate size; however, in the absence of macrosomia, this increased risk may be due to the increase in soft tissue in the pelvis that narrows the pelvic outlet and the negative effect of poor pelvic and abdominal tone on fetal position [17]. In our study, although the high rate of cesarean section was observed in pre-pregnancy overweight or obese women, no significant difference was noted in the rate of emergency cesarean section in relation to pre-pregnancy BMI. This was due to the higher rate of emergency cesarean sections performed due to fetal distress in the underweight group. The underlying mechanism for this effect is unclear, but it can be understood in relation to the observation that pre-pregnancy underweight is related to adverse neonatal outcomes.

Maternal weight gain during pregnancy has recently become a controversial subject. Claire *et al*. reported that excessive gestational weight gain caused long-term consequences to maternal and child body size, but a lower gain increased the risk of SGA. The Institute of Medicine has constantly updated the guidelines for gestational weight gain from 1970 until 2009, demonstrating the difficulty in balancing the risks that are associated with inadequate weight gain against those that are associated with excessive gain. The guidelines suggest an optimal weight gain range that is specific to pre-pregnancy BMIs, and this has been validated in various studies [15,19-22]. The guidelines, however, do not make a recommendation using modified BMI categories based on data from Asian populations. We analyzed perinatal outcomes in relation to the modified BMIs for Asian populations in our study. Gestational weight gain was not noteworthy in the overweight and obesity groups, but in the underweight and normal weight groups, gestational weight gain and perinatal outcomes showed a U-shaped interrelation.

There are some limitations to our study. First, pre-pregnancy body weight was based on self-reported data. Since overweight or obese people tend to understate their weight, it is possible that errors occurred in the process of grouping the pre-pregnancy BMIs. Second, the present study was based on data collected from a single hospital located in an urban area. According to a previous study, excessive weight gain could be prevented in low-income women by intervention for gestational weight gain [23]. The majority of our subjects lives in urban areas and thus had undergone prenatal care, whereupon gestational weight gain could be properly controlled, and this may function as a buffer between gestational weight gain and adverse perinatal outcomes.

Despite such limitations, our study has several strengths. We comprehensively analyzed the influence of pre-pregnancy BMI and gestational weight gain on perinatal outcomes in the same group of pregnant Korean women, and confirmed that pre-pregnancy BMI was a more significant index than gestational weight gain. In addition, our results suggested that neonatal complications increased in underweight women before pregnancy and that inadequate gestational weight gain caused adverse perinatal outcomes. As the obese population has rapidly increased, even in women of childbearing age, underweight pregnant women have been relatively neglected. However, considering that the distribution of the pre-pregnancy underweight population is similar to the overweight and obese population, our results are important in the sense that they indicated that the weight of underweight women should be properly controlled during pregnancy in order to improve the perinatal outcome.

## Conclusions

Pre-pregnancy overweight and obesity are more closely related to adverse obstetric outcomes than excess weight gain during pregnancy. In addition, inadequate weight gain during pregnancy can result in significant complications.

The results of this study are expected to be highly helpful during consultations with women of childbearing age and pregnant women with respect to weight control, as well as to perform clinic-based intervention. Additionally, there is a need to conduct a large-scale multicenter study to compile guidelines for the optimal weight gain range using the modified BMI classification for Asian populations.

## Competing interests

The authors declare that they have no competing interests.

## Authors' contributions

SKC is the first author and participated in the design of the study, acquisition of data, interpretation of data, and drafting of manuscript. IYP conceived the study. JCS is the corresponding author and participated in study design and coordination and drafting of the manuscript. All authors have read and approved the final manuscript.

## Authors' information

Sae Kyung Choi is a clinical fellow of the division of Maternal-fetal Medicine, Department of Obstetrics and Gynecology, Seoul St. Mary's Hospital of the Catholic University of Korea. In Yang Park is an associated professor of the Catholic University of Korea, division of Maternal-fetal Medicine, Department of Obstetrics and Gynecology, Seoul St. Mary's Hospital. Jong Chul Shin is a professor of the Catholic University of Korea, division of Maternal-fetal Medicine, Department of Obstetrics and Gynecology, Seoul St. Mary's hospital. And he is a vice-president of the Korean Society of Maternal Fetal Medicine and a vice-president of the Obstetrics and Gynecology Association for the Southern area of Seoul.

## References

[B1] Vahratian A Prevalence of overweight and obesity among women of childbearing age: results from the 2002 National Survey of Family Growth Maternal Child Health J 2009 13 268 273 10.1007/s10995-008-0340-6 PMC2635913 18415671

[B2] Voigt M Straube S Zygmunt M Krafczyk B Schneider KT Briese V Obesity and pregnancy-a risk profile Z Geburtshilfe Neonatol 2008 212 201 205 10.1055/s-2008-1076995 19085735

[B3] Kanagalingam MG Forouhi NG Greer IA Sattar N Changes in booking body mass index over a decade: retrospective analysis from a Glasgow Maternity Hospital BJOG 2005 112 1431 1433 10.1111/j.1471-0528.2005.00685.x 16167951

[B4] Lu GC Rouse DJ DuBard M Cliver S Kimberlin D Hauth JC The effect of the increasing prevalence of maternal obesity on perinatal morbidity Am J Obstet Gynecol 2001 185 845 849 10.1067/mob.2001.117351 11641663

[B5] Gunderson EP Childbearing and obesity in women: weight before, during, and after pregnancy Obstet Gynecol Clin N Am 2009 36 317 332 10.1016/j.ogc.2009.04.001 PMC2930888 19501316

[B6] Sebire NJ Jolly M Harris JP Wadsworth J Joffe M Beard RW Regan L Robinson S Maternal obesity and pregnancy outcome: a study of 287,213 pregnancies in London Int J Obes Relat Metab Disord 2001 25 1175 1182 10.1038/sj.ijo.0801670 11477502

[B7] Schrauwers C Dekker G Maternal and perinatal outcome in obese pregnant patients J Matern Fetal Neonatal Med 2009 22 218 226 10.1080/14767050902801652 19330705

[B8] Leung TY Leung TN Sahota DS Chan OK Chan LW Fung LW Lau TK Trend in maternal obesity and associated risks of adverse pregnancy outcomes in a population of Chinese women BJOG 2008 115 1529 1537 10.1111/j.1471-0528.2008.01931.x 19035989

[B9] Gale CR Javaid MK Robinson SM Law CM Godfrey KM Cooper C Maternal size in pregnancy and body composition in children J Clin Endocrinol Metab 2007 92 3904 3911 10.1210/jc.2007-0088 17684051 PMC2066182

[B10] Choo V WHO reassesses appropriate body mass index for Asian populations Lancet 2002 360 235 10.1016/S0140-6736(02)09512-0 12133671

[B11] Alexander GR Himes JH Kaufman RB Mor J Kogan M A United States national reference for fetal growth Obstet Gynecol 1996 87 163 168 10.1016/0029-7844(95)00386-X 8559516

[B12] Briese V Voigt M Wisser J Borchardt U Straube S Risks of pregnancy and birth in obese primiparous women: an analysis of German perinatal statistics Arch Gynecol Obstet 2010 in press 10.1007/s00404-009-1349-9 PMC3022146 20098995

[B13] Bhattacharya S Campbell DM Liston WA Bhattacharya S Effect of body mass index on pregnancy outcomes in nulliparous women delivering singleton babies BMC Public Health 2007 7 168 176 10.1186/1471-2458-7-168 17650297 PMC1940246

[B14] Murakami M Ohmichi M Takahashi T Shibata A Fukao A Morisaki N Kurachi H Prepregnancy body mass index as an important predictor of perinatal outcomes in Japanese Arch Gynecol Obstet 2005 271 311 315 10.1007/s00404-004-0629-7 15185098

[B15] Margerison Zilko CE Rehkopf D Abrams B Association of maternal gestational weight gain with short- and long-term maternal and child health outcomes Am J Obstet Gynecol 2010 202 574.e1 8 10.1016/j.ajog.2009.12.007 20132923

[B16] Dempsey JC Ashiny Z Qiu CF Miller RS Sorensen TK Williams MA Maternal pre-pregnancy overweight status and obesity as risk factors for cesarean delivery J Matern Fetal Neonatal Med 2005 17 179 185 10.1080/14767050500073456 16147820

[B17] Magriples U Kershaw TS Rising SS Westdahl C Lckovics JR The effect of obesity and weight gain in young women on obstetric outcomes Am J Perinatol 2009 26 365 371 10.1055/s-0028-1110088 19085680 PMC3065964

[B18] Poobalan AS Aucott LS Gurung T Smith WCS Bhattacharya S Obesity as an independent risk factor for elective and emergency caesarean delivery in nulliparous women- systematic review and meta-analysis of cohort studies Obes Rev 2009 10 28 35 10.1111/j.1467-789X.2008.00537.x 19021871

[B19] Stotland NE Haas JS Brawarsky P Jackson RA Fuentes-Afflick E Escobar GJ Body mass index, provider advise, and target gestational weight gain Obstet Gynecol 2005 105 633 638 10.1097/01.AOG.0000152349.84025.35 15738036

[B20] DeVader SR Neeley HL Myles TD Leet TL Evaluation of gestational weight gain guidelines for women with normal prepregnancy body mass index Obstet Gynecol 2007 110 745 751 10.1097/01.AOG.0000284451.37882.85 17906004

[B21] Kiel DW Dodson EA Artal R Boehmer TK Leet TL Gestational weight gain and pregnancy outcomes in obese women: how much is enough? Obstet Gynecol 2007 110 752 758 10.1097/01.AOG.0000278819.17190.87 17906005

[B22] Cedergren MI Optimal gestational weight gain for body mass index categories Obstet Gynecol 2007 110 759 764 10.1097/01.AOG.0000279450.85198.b2 17906006

[B23] Olson CM Strawderman MS Reed RG Efficacy of an intervention to prevent excessive gestational weight gain Am J Obstet Gynecol 2004 191 530 536 10.1016/j.ajog.2004.01.027 15343232

